# Method of Fuel Injector Diagnosis Based on Analysis of Current Quantities

**DOI:** 10.3390/s22186735

**Published:** 2022-09-06

**Authors:** Krzysztof Więcławski, Tomasz Figlus, Jędrzej Mączak, Krzysztof Szczurowski

**Affiliations:** 1 Faculty of Automotive and Construction Machinery Engineering, Warsaw University of Technology, 05-524 Warsaw, Poland; 2 Faculty of Transport and Aviation Engineering, Silesian University of Technology, 40-019 Katowice, Poland

**Keywords:** fuel injector, fuel system, combustion engine, diagnosing, electrical properties, mechanical

## Abstract

This paper discusses a method of diagnosing electromagnetic valves of injection systems in combustion engines. Based on multiple analyses of electrical quantities occurring in the course of the electromagnetic injector work and physical relationships between them, the quantities have been demonstrated on which the fluctuation of the electromagnetic force in the injector depends. Moreover, the results of its fluctuations have been mapped to the electric quantities controlling the fuel injector’s work. The research has shown that the current and voltage waveforms contain information on electrical properties of the injector coil and its mechanical properties determining the injector’s technical health as well as that of the fuel system.

## 1. Introduction

Electromagnetic valves are widely used in the field of machine and appliance design. Their proper operation influences the work reliability, but also, it affects the environment, including having a negative influence. Ensuring the correct operation of electromagnetic valves requires systematic tests during operation. Developing new, non-disassembly methods for the evaluation of the technical condition of valves is an important direction of scientific work.

Electromagnetic valves constitute popular solutions in injection systems of combustion engines used in the means of transportation. They are applied as executive elements in injection systems in both spark ignition and compression ignition engines.

The research work conducted so far, devoted to injectors and electromagnetic valves, focused on tests in regard to the fuel injector design, development of research methods, and evaluation of the injected fuel stream, but also the emissions of toxic components of exhaust gases. The authors of works [1,2,3] used measurements and analyses of vibrational signals to test fuel injectors and evaluate the quality of the injection process. In work [4], the authors applied conducted tests, a combination of qualitative imaging techniques and quantitative techniques for the determination of the drop size, such as laser diffraction and Doppler anemometry (PDA). The backlight fast imaging techniques and fast radial laser imaging in injector tests were discussed in [5]. Tests of the injected fuel were also conducted in work [6], in which the Schlieren shading technique was used to track the gas phase of the spray during the measurements or the technique of the focused shadowgraph, and Mie scattering was used to track the liquid portion of the spray. The research regarding the effects of fuel injectors and the type of fuel supplied on the emissions of toxic ingredients of exhaust gases can be found in [7,8,9]. The research on diagnosing the elements of fuel injection, including the effect of change of their technical state, can be found in [10,11] and in works [12,13,14,15,16], the research on the effects of sediments on the injector. Mohan B. et al. in [17] discuss the method of measurement based on gauging the momentum flow of the atomized fuel stream from each injector nozzle opening and determining the coefficient of the outflow share from a given opening in relation to the others. 

While analyzing the injector work, the following needs to be noted: The fuel flow is determined using Coriolis mass flow meters [18]. The engine rotational speed signal is also used for this purpose, as well as the system adaptation counter corresponding to changes in the subassemblies, pressure fluctuations in the intake manifold, and fluctuations of the fuel pressure related to dosage [19,20]. Modifications of the injector work are reflected in the combustion process in the engine [21], which translates into a change in the number of toxic substances in the exhaust gases. Additionally, changes in the dosage resulting from the injector’s improper operation while changing its characteristics [22] entail difficulties in starting the engine. The engine controller manages the ratio of the fuel to air supplied to the combustion chamber; it also manages the ignition, throttle position, idle speed, EGR valve operation, and other assemblies, depending on the engine type [23,24]. Each of the assemblies is vital for the functioning of the engine; however, the injector, as a final element in the fuel system, plays a substantial role in the proper combustion process [25,26] and emissions of toxic substances in the exhaust gasses [27]. In turn, the infallibility of the injector work depends in the first place on its efficiency in generating the magnetic flux and on the value of the resultant magnetic force, causing the needle to move. 

The presented information indicates that the observation of the current quantities may enable the control of the fuel flow volume through the injector nozzle [28]. These phenomena have not been directly used so far by scientists in the process of injector diagnosing during the evaluation of their technical state. 

The paper presents a study that aims to determine what measurement quantities the magnetic force in the injector depends on and to relate the effect of that force to the quantities managing the operation of the fuel injector. The authors present the theoretical basis for the change in the current values occurring in the fuel injector and explain the influence of changing the derivative sign on the injector dosage.

Further, the authors’ method for diagnosing the fuel injectors and the characteristic quantities necessary for their application is discussed. Several example tests and analyses of the current signals are presented to illustrate the implementation of the developed test method in the practice of diagnosing the technical condition of fuel injectors in internal combustion engines.

## 2. Magnetic Force

**Figure 1:**
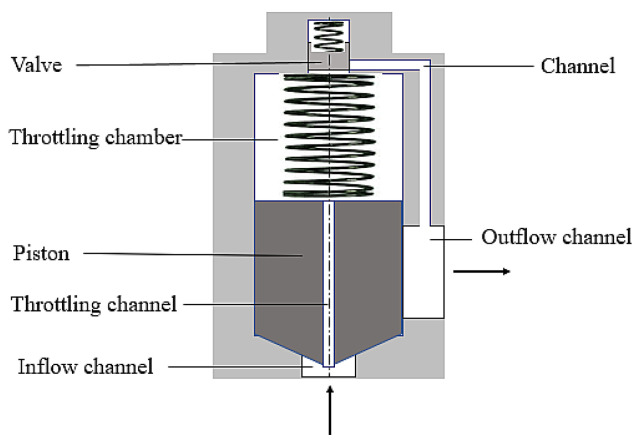
Electromagnetic valve.

An electromagnetic valve applied in the technical context is a working element used wherever control of the liquid or gas flow is required. Its basic element is an electromagnetic coil powered and controlled by means of change in the electric signal. The current flow through the valve coil induces the electromagnetic field, attracting a ferromagnetic element (e.g., a needle), whose movement opens or closes the flow of the specific medium. The needle’s return after the electric current impulse that was shut off is ensured by the spring pressing it against the seat. Figure 1 illustrates an exemplary electromagnetic valve.

A natural property of electromagnetic valves, and thus fuel injectors as well, is the asynchronism of the fuel flow phase relative to the phase of the control impulse. The cause of it is a gradual increase in the magnetic flux generating a magnetic force of appropriate value that will lift the injector needle and, with a certain phase shift, will unblock the flow. 

**Figure 2:**
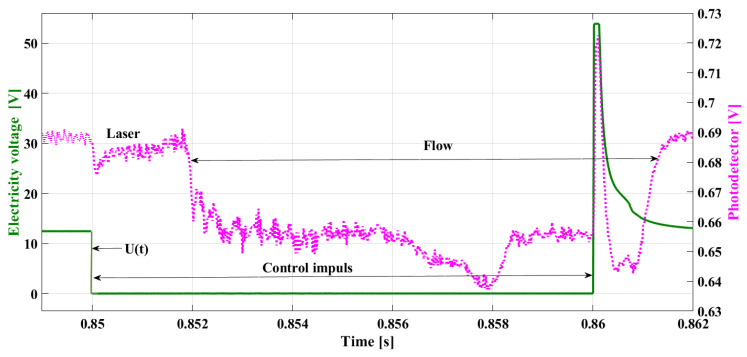
Delay of the fuel flow relative to the impulse controlling the fuel injector.

The value of the delay is determined by the time constant of the injector coil [29,30], which denotes its electromagnetic properties. The shift in the needle stroke is also related to density and fuel pressure, the conditions affecting the needle’s operation. Figure 2 shows a sample asynchronism of the fuel flow (the pink line) relative to the phase of the control pulse (the green line—the electric voltage in the coil) obtained as a result of a test bench experiment. In this research, a laser beam was located under the injector outlet channel. The outflowing stream diminished the voltage at the photodetector, which reflects the real phase of the fuel flow.

Performing the detailed analysis of the presented characteristics allows for the observation that the range of the control impulse is not synonymous with the fuel flow phase. This fact can be taken into consideration in injector control. The possibility of determining the phase of real flow in real-time—in the course of the injector work—and using this information for control would enable the correction of the duration of the control impulse and the flow phase. This information can be used by the ECU to perform its controlling function. 

The accomplishment of this task requires finding a way to determine, in the course of the engine’s operation of the fuel injector, the actual start, and end of the injection. This method may well be the observation of the current parameters of the dosing injector because the current which supplies the injector’s coil is also a control signal. 

**Figure 3:**
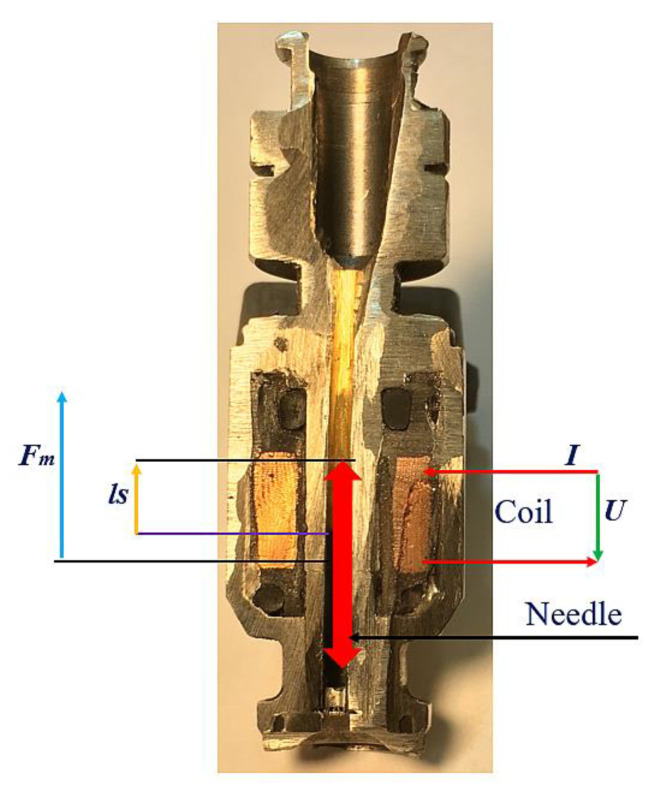
Distribution of ferromagnetic materials in the injector housing.

**Figure 4:**
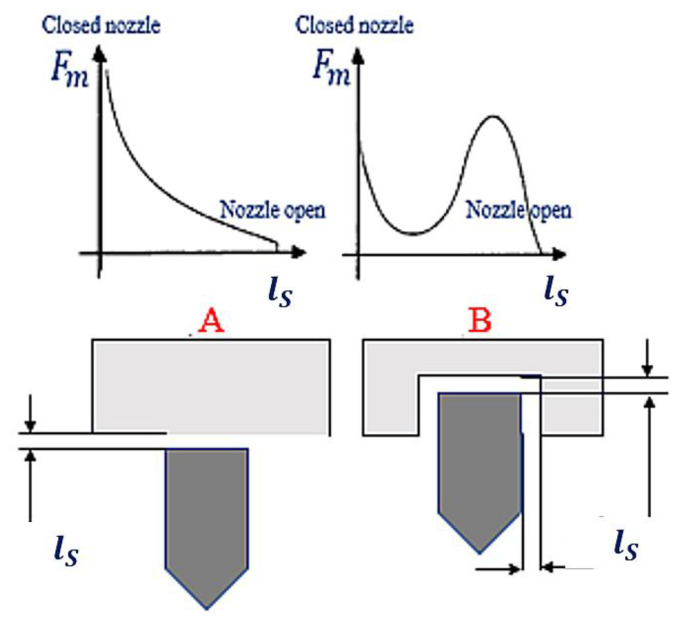
Relationship between magnetic force $F_{m}$ and the size of gap $l_{S}$. These are two cases, A and B.

The movement of the injector’s needle is accompanied by the change in the gap between this needle and the core. To put it more precisely, during its motion, the needle gradually fills the inside of the core (Figure 3 and Figure 4), subject to the increasing power of magnetic attraction [29]. Figure 4 shows the change in the magnetic force $F_{m}$, depending on the size of the gap $l_{S}$. 

**Figure 5:**
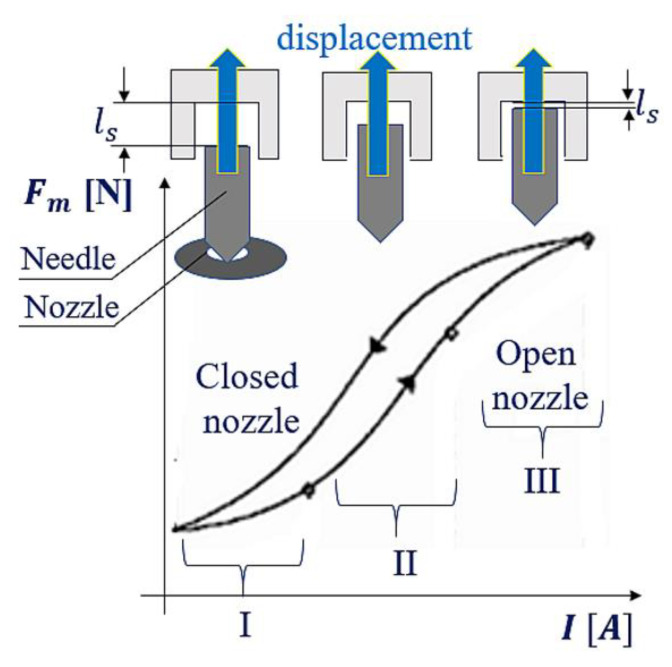
Distribution of magnetic force $F_{m}$, depending on the electric current intensity *I* and the needle retraction into the injector core (gap $l_{S}$ ).

Figure 4 shows that the function of the magnetic force distribution $F_{m}=f\left(l_{S}\right)$ during the needle lifting is not clearly defined. The dependence of the magnetic force $F_{m}$ on the electric current intensity is also ambiguous (the waveform in accordance with hysteresis), which is illustrated in Figure 5. It shows three phases of the increase in the magnetic force $F_{m}$ and the corresponding current intensity *I*. During the first stage, the air gap is at its maximum, hence the maximal magnetic resistance. The magnetic force grows proportionally to the square of the current [30]:

- stage I:

(1) $$F_{m}\sim I^{2}$$

In the second stage, the magnetic force almost linearly depends on the current:

- stage II:

(2) $$F_{m}\sim I$$

In the third stage, the magnetic circuit is in a state of saturation. The increase in current corresponds to a smaller increase in magnetic force than in the previous stages:

- stage III:

(3) $$F_{m}\sim \sqrt{I}$$

**Figure 6:**
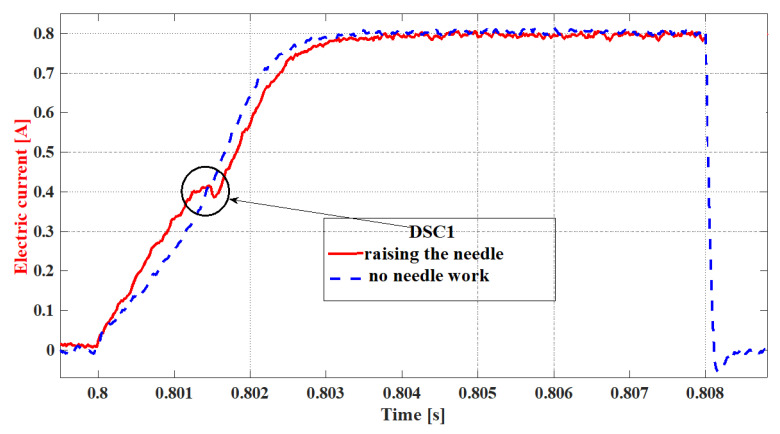
Comparison of the current waveforms of the *RL* circuit, performing the work and non-performing.

**Figure 7:**
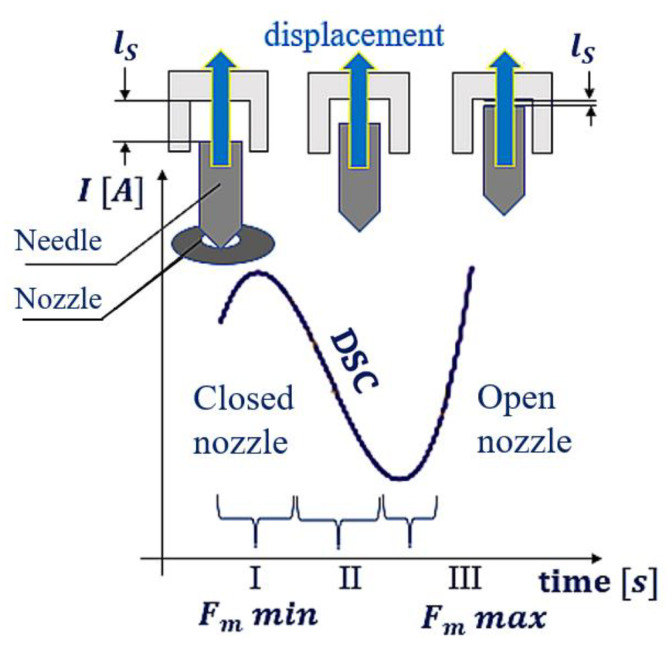
Transient state. The point of the needle lifting with the phases of change in the magnetic force $F_{m}$, gap, and current intensity. Duration: ~100 μs.

The discussed stages (I, II, and III) determine the character of the electric current plot in the transient state (Figure 6—the round, black indicator at the electric current waveform). In Figure 7, the transient ranges (I, II, and III) are assigned to the stages of change in the magnetic force $F_{m}$ and electric current intensity *I*.

The magnetic force $F_{m}$ is a derivative of the energy generated in the coil as a result of the action of the current. Current changes in the RL circuit are described by Kirchhoff’s law:

(4) $$RI+L\frac{dI}{dt}=\varepsilon$$

where: 

*R*—resistance [Ω],

*L*—inductance [H], 

*Ε*—electromotive force [V].

The magnetic force $F_{m}$, lifting the needle, is a derivative of energy accumulated in the coil, and the shift related to its operation is a needle displacement ($l_{s})$ (illustrated in Figure 4, Figure 5 and Figure 7):

(5) $$F_{m}=\frac{dE_{m}}{dl_{s}}=\frac{-N^{2}}{{\left(\frac{l_{c}}{\mu S}+\frac{l_{s}}{\mu_{0}S}\right)}^{2}\mu_{0}S}\;\;\frac{I^{2}}{2}$$

or:

(6) $$F_{m}=\frac{dE_{m}}{dl_{s}}=\frac{-L^{2}}{\mu_{0}S}\;\frac{I^{2}}{2}$$

The constant quantities occurring in Equation (6) are: the number of turns (*N*), the vacuum magnetic permeability ($\mu_{0}$), and the surface of the coil cross-section (*S*). What ensues is that the magnetic force is a function dependent on the following variables (7):

- electric current intensity;
- relative permeability (of the core material along with the gap, $\mu =\mu_{0}\mu_{R}$);
- gap length (depth of the needle insertion into the core $l_{s}$);
- inductance.

(7) $$F_{m}=f\left(I,\;\mu_{R},R_{M},\;l_{s},\;L\right)$$

The analyses conducted in this section enabled the determination of the physical relationships influencing the occurrence of the range of “the sign change of the waveform derivative” shown in Figure 7, as well as establishing what affects “the sign change of the waveform derivative”.

## 3. *DSC*—Derivative Sign Change

A crucial factor in the analysis of the injector operation is the determination of the influence the sign change of the waveform derivative, denoted by *DSC* (Derivative Sign Change), has on the injector dosage, as well as establishing why the area of the dosage marked in the characteristic plots as *DSC1* can be observed at the given value of the current intensity with the specific time delay. The sign change of the waveform derivative *DSC*, defining the displacement of the injector needle, thus, requires reconfiguration of the quantities from Equation (7).

The possibility of determining the magnetic force from the current values and the joint dependence of both parameters allows the conclusion that the mechanics of the needle displacement will be reflected in the electric current waveform. Figure 6 shows two current waveforms that enable the proving of this assumption. The blue line denotes the current intensity in the injector coil, whose needle does not perform any work (because of being blocked). A steady increase in the current intensity can then be observed. The red line describes the waveform of an injector with a regular dosage. Due to the displacement of the injector needle, there occurs a *DSC1* range (marked with a black, round symbol). In the *DSC1* range, a local stoppage in the current intensity growth occurs, and there is a decrease in this value in spite of the circuit coil not reaching the state of saturation.

**Figure 8:**
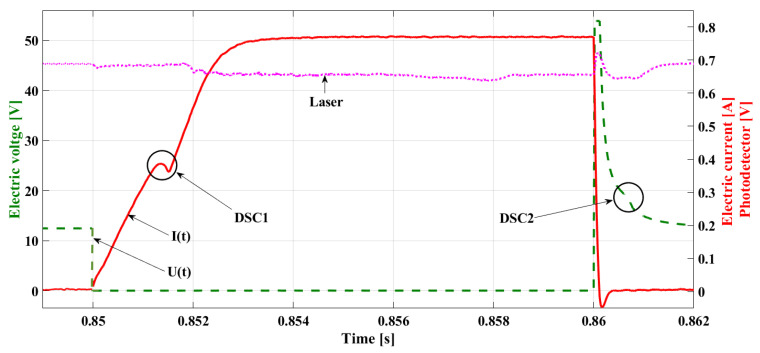
The current waveform of the dosing injector with marked ranges of needle lifting and dropping, *DSC1*, and *DSC2*.

In the waveform of the electric current describing the injector work (Figure 8), there are two such ranges to be observed, connected with the needle displacement, cutting the flow off. The starting point of the needle lifting is described by the first *DSC* (*DSC1*)—the subsequent changes of the sign of the current intensity derivative (8). After cancelling the control impulse, the dropping of the needle is described by the second *DSC* (*DSC2*)—which means the change in the derivative of the current–voltage (9) during its deterioration.

(8) $$I\left(t\right)=\left\{\begin{array}{c} \frac{dI}{dt_{n}}>0\\ \frac{dI}{dt_{n+1}}=0\\ \frac{dI}{dt_{n+2}}<0\\ \frac{dI}{dt_{n+3}}=0\\ \frac{dI}{dt_{n+4}}>0 \end{array}\right.$$

(9) $$U_{L}\left(t\right)=\left\{\begin{array}{c} \frac{dU_{L}}{dt_{n}}<0\\ \frac{dU_{L}}{dt_{n+1}}=0\\ \frac{dU_{L}}{dt_{n+2}}<0 \end{array}\right.$$

where: 

*t*—time [s];

$U_{L}$—electric voltage (on the inductance) [V].

Figure 8 shows a current waveform with the *DSC1* marked on the line denoting the increase of the current intensity and *DSC2* on the line denoting the decrease in voltage. Additionally, in Figure 8, the pink line represents the fuel flow and is determined by the drop in voltage from the photodetector after the laser light was disturbed during measurements. The delay in the occurrence of the flow is visible, relative to the symptoms of the needle stroke determined by the current changes (*DSC1*). This phenomenon results from the fuel density and gradual increase of the stream. The record of the growing current intensity in the *RL* circuit in the case of the work not being performed (e.g., needle lifting) is a steady increase in the current intensity up to its maximal value, limited by the coil resistance and electromotive force of the supply source in accordance with Kirchhoff’s Equation (10), as shown by the blue line in Figure 6.

(10) $$I\left(t\right)=\frac{\varepsilon_{0}}{R}*\left(1-exp\left(-\frac{R}{L_{1,2}}*t\right)\right)$$

The current waveform of the dose-emitting injector is not depicted by Kirchhoff’s equation in its original form (10). The occurrence of the forces counteracting the needle lifting (11) causes the emergence of *DSC1*, and Kirchhoff’s equation, reflecting such an increase in electric current intensity, needs to be completed with a factor taking into account the influence of the forces being overcome:

(11) $$F_{m}>F_{p}+F_{i}+F_{f}+F_{s}$$

where:

$F_{p}$—the force resulting from the fuel pressure;

$F_{i}$—inertial force;

$F_{f}$—frictional force;

$F_{s}$—the force of the spring.

Kirchhoff’s equation, a result of which the value of the current intensity $F_{s}$ will be obtained, corresponding to the range of the needle lifting, is determined by the equation below, where the factor $f_{press}$ diminishes the resultant value of the electric current intensity:

(12) $$I\left(t\right)=f_{press}*\frac{\varepsilon_{0}}{R}*\left(1-exp\left(-\frac{R}{L_{1}}*t\right)\right)$$

where $f_{press}$ is the factor taking into account the operation of the forces from Equation (11).

The performed analyses allow for the conclusion that in the injector current waveform, there is information not only on the electric properties of the injector coil but also regarding the injector’s mechanical properties, determining its efficiency, as well as that of the fuel system [31].

## 4. Method of Diagnosing Fuel Injectors and Characteristic Quantities

Due to the fact that the character of the increase in the injector current intensity reflects mechanical and electric phenomena at the point of the needle lifting in the dose-emitting injector, the assumption has been made, creating the method of diagnosing fuel injectors, that information on the value of the current intensity at the point of the needle lifting and on the decrease in the voltage at the point of the needle dropping will be used in the process of the examination of the technical state [32]. 

**Figure 9:**
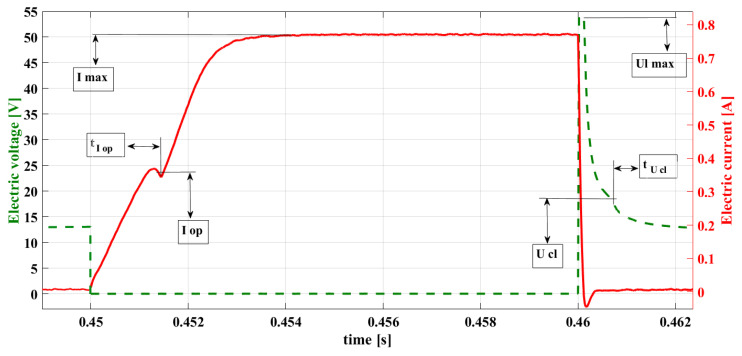
Characteristic points of a sample current waveform.

On the basis of the significant amount of laboratory tests conducted for many different fuel injectors (indirect injection, gasoline, and gas injectors), the characteristic points of the current waveform for the dose-emitting injector were determined (Figure 9):

- $I_{op}$—the electric current intensity at the point of needle lifting;
- $t_{I_{op}}$—the phase of the occurrence of value $I_{op}$, *DSC1*;
- $I_{max}$—the value of the electric current intensity in the steady state (maximum);
- $U_{L,max}$—the value of electric voltage at the maximum of the induction peak;
- $U_{cl}$—the electric voltage at the point of the needle dropping;
- $t_{U_{cl}}$—the phase of *DSC2*’s occurrence.

The diagnostic research conducted on the basis of the observation of the characteristic points of the current waveforms enables verification of the injector in the course of the test bench tests on the working engine in real-time. The observation of the values of $I_{op}$ and $t_{I_{op}}$ enables the determination of the beginning of the real fuel flow (the stroke of the injector needle), control of the fuel pressure before the injector, and detection of the needle blockage; such an observation also verifies the electric efficiency of the circuit. 

The following points 1–3 indicate the way of conducting the analysis of current signals that would reflect the principles of the developed method for diagnosing the fuel injectors. On the basis of the bench tests performed on the real injectors, the abnormalities in the electromagnetic injector operation that are possible to be diagnosed have been determined. Sample tests were conducted on the basis of the registered characteristics of the injector, presented in Figure 9.

*The way of performing the analysis of the current signals:*

1. The maximal value of the current in the steady state $I_{max}$ should be determined by the results from the value of the resistance of the injector coil *R* = 14.8 Ω and from the supply value of the current source (11.6 V). The calculation based on the Ohm law renders the maximal intensity $I_{max}\;$= 0.78 A (Figure 8).
  The observation of this value allows for the evaluation of the invariability of the circuit resistance and the value of the electric voltage supply.
2. The value of the electric voltage at the maximum induction peak ($U_{L,max}$) results from the maximal value of the current intensity *I* = 0.78 A, the speed of its deterioration, and inductance of the injector coil core *L H*:
  (13) $$U_{max}=\frac{dI}{dt}*L$$
  The observation of this value allows for verification of the efficiency of the electric circuit (no short circuit).
3. Reconfiguration of the value $U_{cl}$ and phase $t_{U_{cl}}$ indicates the failure of the return spring of the injector needle, the needle blockage, or the occurrence of the short circuit.

## 5. Sample Research Results and Their Analysis

**Figure 10:**
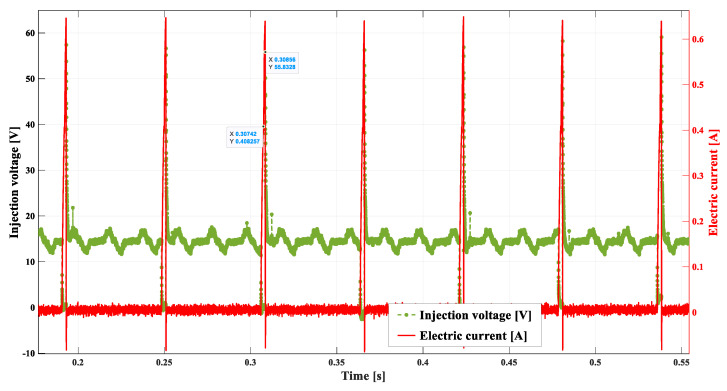
Example representation of cyclic injections at 2000 rpm.

Figure 10 shows a single control impulse of the injector at the rotational speed of 800 rpm, i.e., while the engine is idling. The duration of the injection, in this case, amounts to approx. 0.0022 s.

The model values of the current intensity and electric voltage at the vital points of the injector work, constituting the range of limit values determining the proper condition of the fuel injector and the fuel system, are, respectively [32]:

- *DSC1* = 0.40729 A, the point of needle lifting;
- $t_{delay}\;$ = 0.00136 s, the delay in needle lifting;
- $U_{L}max$ = 55.46 V, the induction peak after cancelling the impulse;
- $t_{inj}$ = 0.0022 s, the duration time of the injection set by the ECU;
- $t_{real}$ = 0.0014 s, real-time of the fuel flow.

**Figure 11:**
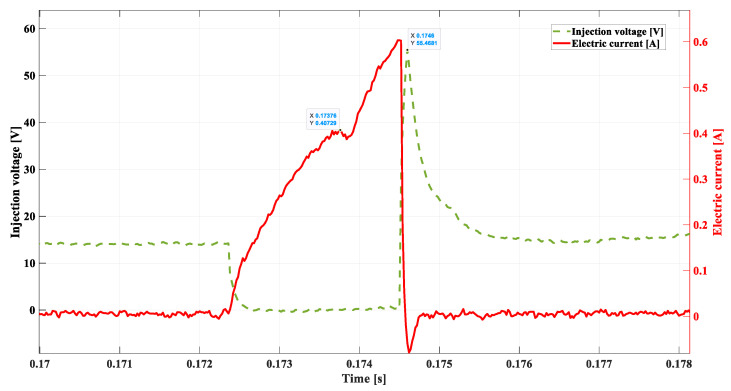
Real waveform of electric voltage and current at 800 rpm.

The waveform of the electric voltage at the point *t* = 0.1752 (Figure 11) allows for the observation of the slight increase in the voltage in the phase of its deterioration—point *DSC2*. 

This point is of diagnostic significance, but also, its phase is crucial for the proper control. Observation of this change is difficult but offers a possibility of using its relationship to the maximal value of the inductive voltage peak $U_{L}max$. Changes in the value and in phase *DSC2* take place simultaneously, with changes in the $U_{L}max$, which are easily observed due to the big differences occurring in the case of the emergence of anomalies. Alongside this data, there will be the time for the real fuel flow indicated ($t_{real}$), calculated on the basis of the points determined during the experiment with the photodetector (Figure 2 and Figure 8).

**Figure 12:**
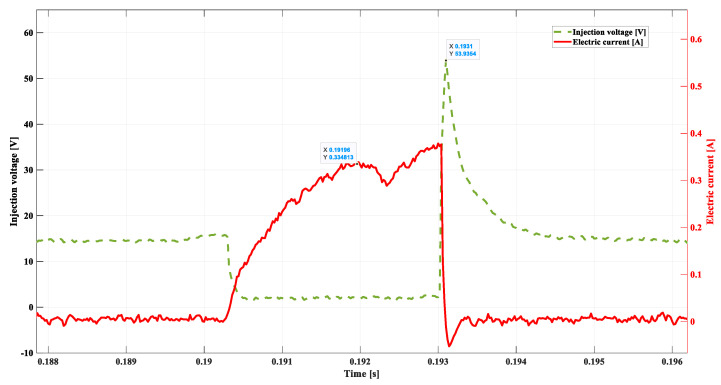
Voltage and current waveform. Resistance 10 Ω, 800 rpm.

Figure 12 shows the voltage–current waveform during the simulation of the failure of the connector or the supply bundle. The damage was simulated by means of modification in the injector coil supply through the connection of the 10 Ω resistor, in series, with the power supply cord. 

These tests ensued (Figure 12) that changes at the characteristic points of the voltage–current waveforms determining the injector state amount to:

- *DSC1* = 0.3348 A;
- $t_{delay}$ = 0.00156 s;
- $U_{L}max$ = 53.93 V;
- $t_{inj}$ = 0.0027 s;
- $t_{real}$ = 0.0014 s.

The analysis of the emerging changes at the characteristic points leads to the conclusion that evident differences for such a type of damage occur in regard to point *DSC1*. As predicted (Ohm’s law), the value of this point has decreased (A). The delay in the time of needle lifting $t_{delay}$ has increased, and the controller reacted by extending the injection duration time ($t_{inj}$). The ECU reaction allows for maintaining the unchanged dose ($t_{real}$) in spite of the modification of the injector supply.

**Figure 13:**
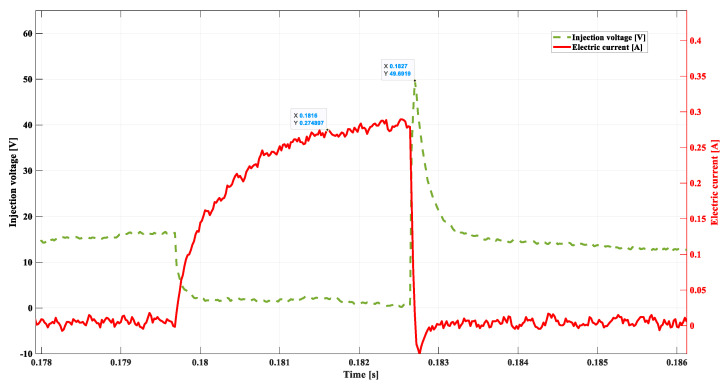
Including the resistance of 20 Ω, 800 rpm.

In the subsequent experiment, the resistance of 20 Ω was added in series within the injector coil supply cord; the results are shown in Figure 13. 

The observation can be made that there exists a certain trend in changes in the recorded parameters. The compilation of the significant values obtained in this experiment is presented below:

- *DSC1* = 0.274 A;
- $t_{delay}$ = 0.00195 s;
- $U_{L}max$ = 49.69 V;
- $t_{inj}$ = 0.003 s;
- $t_{real}$ = 0.0014 s.

The value of the current intensity at *DSC1* has decreased. The result of a smaller value of the current is the longer time of its growth to achieve a proper value of the magnetic stream around the injector coil. This is the reason for increasing the time $t_{delay}$, and as a consequence, the controller reacts by means of increasing the injection time duration ($t_{inj}$). In such a case, the unchanged fuel dose is maintained in spite of the modification of the injector supply ($t_{real}$).

**Figure 14:**
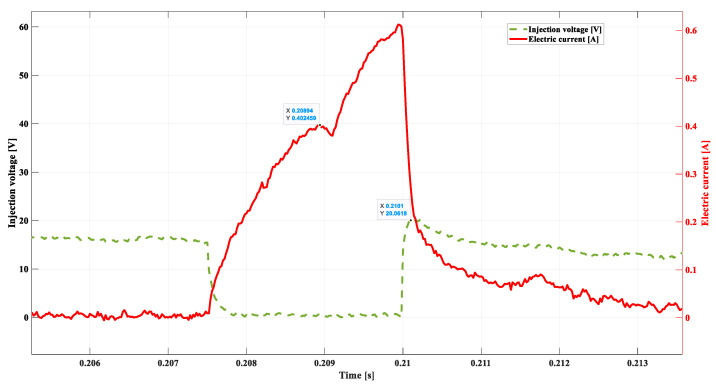
Short circuit using a 20 Ω resistor, 800 rpm.

In the subsequent experiment, a short circuit was introduced between the electric wires supplying the injector coil with a resistance of 20 Ω. The results of the recorded voltage–current waveforms are shown in Figure 14. 

This method of short-circuit simulation was selected as safe, not inducing damage to the elements of the electrical system. This modification did not block the injector dosage yet critically changed the waveform of the electric voltage in the coil. The inductive voltage peak after cancelling the control impulse reached the value of 20.06 V, which constitutes 36% of this quantity in the model waveform. The compilation of the significant values obtained in this experiment is presented below:

- *DSC1* = 0.4024 A;
- $t_{delay}$ = 0.0014 s;
- $U_{L}max$ = 20.06 V;
- $t_{inj}$ = 0.0025 s;
- $t_{real}$ = 0.0029 s.

A controlled short of this value results in a subsequent return dropping of the needle. A consequence of this action is an increase in the real injection time ($t_{real}$). Observation of the *DSC2* point is difficult, yet its correlation with the value $U_{L}max$ allows for the unambiguous recognition of such damage.

**Figure 15:**
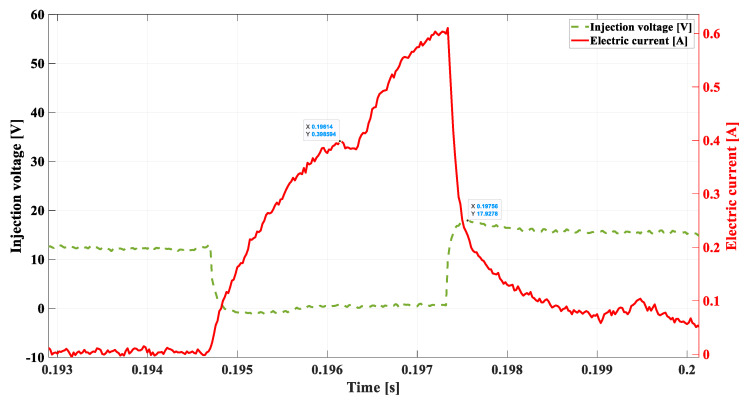
Short circuit using a 10 Ω resistor, 800 rpm.

In the subsequent experiment, a short circuit was introduced in the injector supply circuit with a resistance of 10 Ω (Figure 15), which affects a supply circuit to a greater extent than a short circuit at a value of 20 Ω. 

The analysis of the recorded waveforms renders that the trend in the obtained results is in accordance with the introduced modification. The compilation of the obtained values is presented below:

- *DSC1* = 0.3985 A;
- $t_{delay}$ = 0.00195 s;
- $U_{L}max$ = 17.92 V;
- $t_{inj}$ = 0.003 s;
- $t_{real}$ = 0.0033 s.

The controlled short circuit of a value greater (lower resistance = 10 Ω) than in the previous experiment (20 Ω, results shown in Figure 14) results in an even more delayed return lowering of the needle. A short circuit in an installation of such a value ensures that the time of increasing the current required to lift the needle ($t_{delay}$ = 0.00195 s) is extended. The phase shift of the fuel flow takes place. The controller reacts by extending the injection time ($t_{inj}$ = 0.003 s), which in combination with a subsequent dropping of the needle, results in the extending of the real-time of the fuel flow ($t_{real}\;$= 0.0033 s).

Table 1 shows the compilation of the results obtained in the conducted experiments, where the red print indicates vital changes. The increase in the resistance of the connector ensues the decrease of the *DSC1* value; the controller extends the time of injection ($t_{delay}$), maintaining the unchanged fuel dose. The controlled short circuit in the injector coil supply causes the increase in the time of the fuel flow, which is not a result of the injection time preset by the ECU, but a consequence of a delayed fuel flow shutdown. 

The experiment conducted with the use of the ECOTEC X18XE engine has proved that the observation of parameters (the model parameters) shown in Table 1 allows for the determination of the state of the supply system and the fuel injector. 

## 6. Discussion

The magnetic force and forces counteracting the lifting of the needle affect the shape of the current waveforms. If it were not for the work performed by the needle, dependent on its lifting resistance, the current waveform would develop according to the curve of the exponential character without DSC1 (Figure 6, the blue line). The change of the derivative of the observed current waveform is inextricably linked to the work performed by the magnetic force $\left(F_{m}\right)$ generated by the coil as a result of the flow of electric charges. 

Occurrence of the DSC1 results from several mutually dependent phenomena. The work performed by the injector consists of a displacement of the needle, i.e., a change in the placement of the ferromagnetic material in the electromagnetic field of the core. Shifting of the needle means a change in the distance $\left(l_{S}\right)$ between the needle and the center of the electromagnetic field, which causes an increase in the magnetic permeability $\left(\mu_{R}\right)$, a decrease in the magnetic resistance $\left(R_{m}\right),$ and a growth of the core inductance $\left(L\right)$. Reconfiguration of these quantities in a very short time (~100 μs) renders, as a result, the change of the sign of the waveform derivative $\left(I\left(t\right)\right)$. 

The value of current intensity, at which the change of the derivative sign takes place, results from the degree to which the forces from Equation (11) counterbalance each other. On the left side of the inequality (or equation, depending on the phase) is the electromagnetic force resulting from the value of inductance and from the value of the current flow. On the right side of the equation, there are the forces counteracting the lifting of the needle (11). The balance of these counteracting forces influences the value of the current intensity at the point of the change of the waveform derivative sign $\left(DSC1\right).$ The greater the values of the forces $\left(F_{p,\;i,\;f,\;s}\right)$, the higher on the ordinate axis the DSC1 is located. Change in the balance of these forces, apart from the shift in the value of the current intensity at the point of the needle lifting $\left(DSC1\right)$, also changes the phase of its emergence.

The conducted laboratory experiments have proved that the characteristics of the DSC1 occurrence, depending on the parameters of the injector control, can be developed. Due to the constancy of forces such as the inertial force of the needle $F_{i}$, the frictional force of the needle acting against the guide sleeve $F_{f}$ and the spring force $F_{s}$, such a characteristic would depend on the variable force and the greatest of the above-mentioned forces, i.e., the force resulting from the pressure difference upstream and downstream of the injector ($\Delta_{p}$). 

Implementation in the controller in charge of the engine work (and of the injector) of the module recognizing the change of the waveform derivative in the specified ranges of the current waveform will enable obtaining of the automatic tool detecting damage in the fuel system and the possibility of correcting the length of the control impulse and the injection phase in the current time.

## 7. Conclusions

The fuel injector is an executive element particularly susceptible to damage. Directly generating the fuel dose and, as a final element of the fuel system, it influences to a great extent the proper functioning of the engine, as well as the quality of the fumes from the exhaust system. The fuel injector plays an important role in engine operation in compliance with ecological guidelines; it also affects the safety of vehicle users. The monitoring of the injector operation is vital, particularly if it can be achieved in such a way that the damage would be detected at the early stage when its negative effects are not yet to be felt. 

The analysis of the current waveforms, especially the values and the phase of the DSC occurrence, provides basic information on the course of the injection and the quality of the injector work. The level and the phase shift of the DSC constitute the diagnostic determinants of the dose-emitting injector. The change of any of the current waveform parameters is reflected in this short range of the current waveform. Apart from the parameters of the injector control and its efficiency, it also contains information on the mechanical parameters of the injection system. This information can be used in the diagnostics of the injection system and in injector control. Knowledge of the effect the determinants of the process of the injector control have on the change of the value and phases of the DSC allows for the developing of the diagnostic classifiers of the system, enabling the determination of the emergence of the injector damage. In this paper, the authors have indicated that the current parameters provide precise information; thus, their observation and the development of the characteristic correlating the type of failure with the change of the value and phase of the DSC allows for verification of the system state in both the bench tests and during the engine operation. Using the method proposed in this paper enables the detection of both typical mechanical failures, as well as electrical, even at an early stage of development.

## Figures and Tables

**Table 1:** Compilation of the most significant results from Figure 11, Figure 12, Figure 13, Figure 14 and Figure 15.

	Figure 11	Figure 12	Figure 13	Figure 14	Figure 15
DSC1	**0.40729 A**	0.3348 A	0.274 A	0.4024 A	0.3985 A
$t_{delay}$	**0.00136 s**	0.00156 s	0.00195 s	0.0014 s	0.00195 s
$U_{L}max$	**55.46 V**	53.93 V	49.69 V	20.06 V	17.92 V
$t_{inj}$	**0.0022 s**	0.0027 s	0.003 s	0.0025 s	0.003 s
$t_{real}$	**0.0014 s**	0.0014 s	0.0014 s	0.0029 s	0.0033 s

Red color shows changes essential for the diagnosis of the injector (extreme); Model values are marked in bold.

## Data Availability

Not applicable.
